# Experimental Study on Residual Stress and Deformation Control During Machining of TC18 Titanium Alloy Long Axis

**DOI:** 10.3390/ma18122788

**Published:** 2025-06-13

**Authors:** Xiangyou Xue, Dongyan Shi, Liang Zhao

**Affiliations:** 1 College of Mechanical and Electrical Engineering, Harbin Engineering University, Harbin 150001, China; shidongyan@hrbeu.edu.cn; 2 Shenyang Aircraft Industry (Group) Co., Ltd., Shenyang 110850, China; zhaoliang.0403@gmail.com

**Keywords:** TC18, residual stress, turning, heat treatment, deformation control, chrome plating

## Abstract

The residual stress induced during the processing of titanium alloy materials can significantly influence the deformation control of precision-machined workpieces, especially for workpieces characterized by low stiffness and high-precision requirements. In this study, TC18 titanium alloy forgings with a dense structure were manufactured via forging. By conducting turning and heat treatment experiments on the workpiece, the distribution and evolution of residual stress and the deformation characteristics of TC18 titanium alloy on slender shafts were systematically investigated under different turning and heat treatment conditions. Based on the experimental results, the effects of the turning parameters, including feed rate, cutting speed, cutting depth, and axial thrust force of machine tool center, on workpiece deformation were quantitatively analyzed, and an optimal heat treatment strategy was proposed. The findings indicate that between-centers turning is recommended to control workpiece deformation. Optimal turning parameters include a cutting speed of 640–800 r/min, a feed rate of 0.05–0.1 mm/r, a cutting depth of 0.1 mm, and a thrust force of the center set to 10% of the rated value, resulting in minimal deformation and superior surface quality. In addition, during the heat treatment annealing of slender shaft titanium alloys, residual stress is effectively eliminated at temperatures ranging from 640 to 680 °C with a holding time of 1–3 h. Furthermore, the vertically fixed placement method during heat treatment reduced deformation by approximately 50% compared to free placement. These results provide valuable insights for optimizing machining and heat treatment processes to enhance the dimensional stability of titanium alloy components.

## 1. Introduction

Owing to its high strength-to-density ratio, titanium alloys are widely used in aerospace and military applications [1]. Particularly, TC18 titanium alloy, a high-strength near-β titanium alloy developed by the Soviet Union in the 1970s, features a nominal composition of Ti-5Al-5Mo-5V-1Cr-1Fe [2,3,4,5]. With its composition approaching that of α + β dual-phase titanium alloys, TC18 combines the performance characteristics of both α + β and β titanium alloys. The annealed strength of TC18 reaches levels comparable to solution-treated TC4 alloys, exceeding 1080 MPa, making it particularly suitable for critical structural components in aerospace weapon systems [6,7,8]. These components are typically manufactured through forging and annealing processes to enhance their strength, toughness, corrosion resistance, and fatigue performance. However, the associated residual stresses tend to be gradually released during machining, leading to significant workpiece deformation [9].

The generation of residual stress in a workpiece is caused by various factors. Firstly, it is greatly influenced by the geometric structure. In the forging process of complex workpieces, the plastic deformation caused by material accumulation is uneven, resulting in uneven stress distribution; Secondly, during the heat treatment process, the change in temperature is dramatic, and the cooling rates of different parts of the workpiece are different, resulting in an uneven distribution of thermal stress. Another factor that affects residual stress is the inherent properties of the material itself, and the distribution of residual stress in the final stage is influenced by both the release of residual stress in the high-temperature stage and the accumulation of residual stress in the low-temperature stage. Finally, during the mechanical processing of the workpiece, the workpiece will be cut, ground, and subjected to special processing such as sandblasting, heat treatment, and plating, and the residual stress will be generated on the surface of the workpiece [10]. The presence of residual stress has a significant impact on precision-machined workpieces; especially when the workpiece has low stiffness, such as thin-walled parts or slender shafts, the residual stress will increase the difficulty of achieving deformation control of the workpiece, which leads to it being out-of-tolerance or scrap. At present, there are many studies on the cutting and residual-stress testing of titanium alloys, but mainly focused on titanium alloys such as TC4 [10,11,12,13]. The residual-stress detection after the cutting process is mainly divided into destructive and non-destructive methods, with X-ray detection being the most widely used in non-destructive testing [14,15,16].

Currently, research on residual stress and deformation control during precision machining of low-stiffness titanium alloy long-axis parts remains limited, especially for TC18 titanium alloy materials. Therefore, this work systematically investigated residual stress and deformation control in the precision machining process of TC18 titanium alloy slender shafts. To solve the serious deformation problems of slender shaft workpieces during machining and annealing, we optimized cutting and heat treatment methods based on residual-stress variation and deformation characteristics, and established a reasonable deformation control method for the precision machining of TC18 slender shaft workpieces.

Residual stress refers to the self-equilibrating internal stress that persists within a component in the absence of external loading. It is typically categorized into two distinct types: the first type is inherently present within the material during its manufacturing process, whereas the second type is induced on the workpiece surface during subsequent processing. Prior to machining, the resultant force and moment on any cross-section of the workpiece must satisfy equilibrium conditions, as described by:

∫δdA=0

∫dM=0

During the cutting process, the material removal rate varies significantly across different cross-sections along the axial direction, particularly in long-axis die forgings. The pre-existing deformation of the billet, resulting from forging and heat treatment processes, further complicates the machining behavior. As material is progressively removed, the non-uniform release of internal stresses within the cutting layer occurs. Moreover, the tool–workpiece interaction induces additional stresses on the machined surface. Concurrently, structural alterations lead to variations in workpiece stiffness, causing the workpiece to undergo deformation until reaching a new equilibrium state. This phenomenon explains the deformation mechanism induced by residual stresses during machining.

## 2. Material Preparation and Testing

### 2.1. Long-Axis Structure and Processing Characteristics

**Figure 1:**
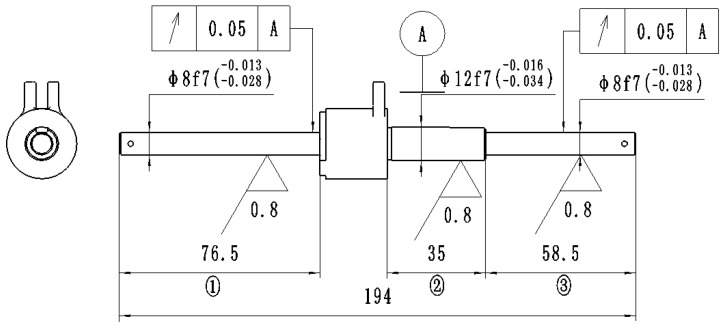
Structural diagram of long-axis workpiece.

As shown in Figure 1, the slender shaft workpiece measures 194 mm in total length. The manufacturing process involves die blank processing, chrome plating, and grinding. After grinding, the three journal diameters from left to right are φ8f7, φ12f7, and φ8f7 with IT7 tolerance grade. The chromium coating thickness should range from 21 to 48 μm with uniform distribution, free from localized detachment. The post-grinding requirements include radial runout ≤ 0.05 mm and surface roughness Ra ≤ 0.8 μm.

The conventional manufacturing process for processing such workpieces includes forging, annealing, making top holes, machining (turning, milling, grinding), incomplete vacuum annealing, sandblasting, milky white chromium coating, vacuum diffusion annealing, hard chromium coating, precision grinding, and final removal of the top. In order to control the deformation of the workpiece, it is necessary to strictly control the cutting scheme when using this process flow. In order to meet the requirements of coating and runout, the pre-plating and post-plating dimensions should be strictly designed. Based on the theoretical calculations, the maximum runout after electroplating should not exceed 0.08 mm. When considering clamping tolerances, according to experience, the runout should be less than 0.05 mm.

### 2.2. Materials Preparation

In this study, TC18 titanium alloy die forging was selected as the processing blank material due to its dense microstructure and excellent mechanical properties. The nominal chemical composition of this alloy is Ti-5Al-5Mo-5V-1Cr-1Fe, with its detailed chemical composition presented in Table 1.

**Figure 2:**
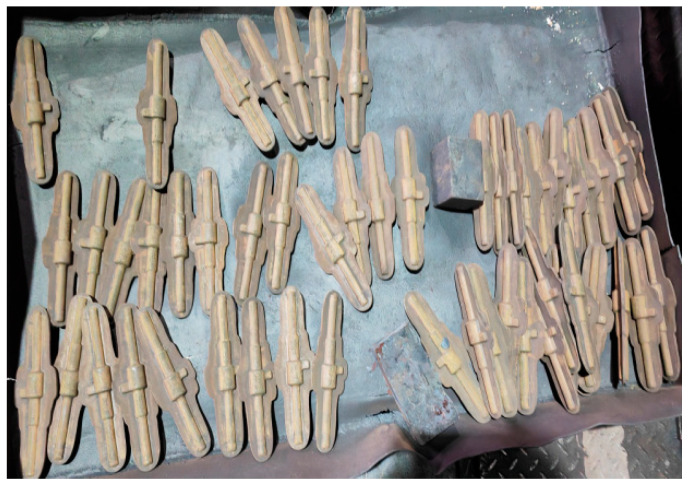
Structure of forging blanks.

**Figure 3:**
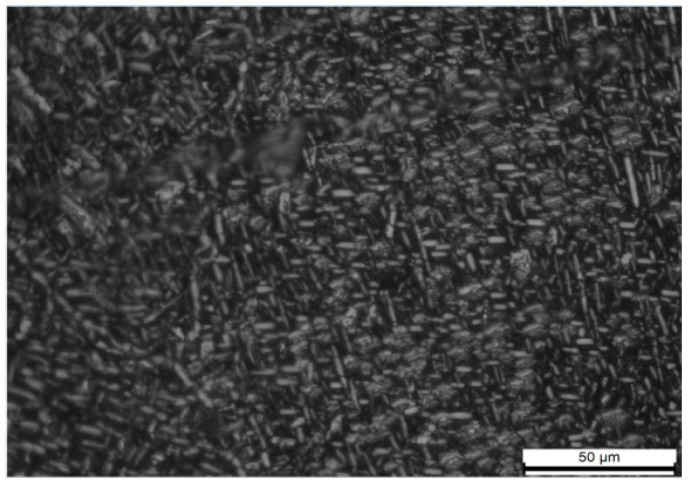
Uniform microstructure of the forging matrix.

Initially, the billet was forged with a radial allowance of 3 mm and **an** axial allowance exceeding 10 mm per side, as shown in Figure 2. Following forging completion, the billet underwent material acceptance testing according to the specified criteria before being approved for use. Microstructural characterization revealed a uniform and dense microstructure, as shown in Figure 3.

### 2.3. Experimental Setup

**Figure 4:**
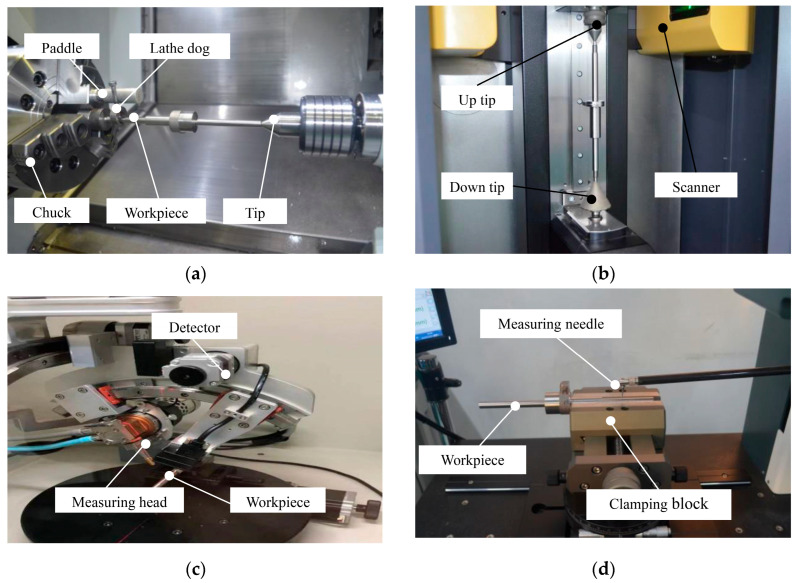
(**a**) Turning clamping method; (**b**) optical detection of radial runout; (**c**) residual-stress measurement; (**d**) surface roughness detection.

To investigate the residual stress and deformation of the workpiece after precision machining and annealing, 9 sets of TC18 titanium alloy forging samples were subjected to turning operations, with the cutting parameters summarized in Table 2. The turning process was performed on a high-precision CNC turning center (Hardinge Machine Tool, Model T51) (Elmira, New York, NY, USA), featuring high automation and machining accuracy. CURIE5885M cutting fluid was employed during machining. Between-centers turning was adopted during processing, as shown in Figure 4a. The maximum radial runout of the workpiece outer diameter was measured using a Swiss Danqing optical shaft scanner (Lausanne, Switzerland) (Figure 4b). Residual stress was analyzed using a Chinese-made XL-640 X-ray stress analyzer (Shanghai, China), which determined surface residual stress via X-ray diffraction (Figure 4c). Surface roughness was evaluated using a profilometer (Xi’an, China), as shown in Figure 4d.

Prior to rough turning, facing was performed on the end face, and center holes were drilled at both ends of the workpiece with a minimum depth of 2 mm and a 60° angle included. The surface roughness of the conical holes was controlled below Ra 6.3 μm to minimize runout caused by center positioning and improve positioning accuracy. Following outer diameter finishing, the deformation and residual stress distribution of the workpiece were measured. Subsequently, the workpiece underwent incomplete annealing at 640 °C for 1.5 h, after which deformation and surface residual stress were re-evaluated.

## 3. Results and Discussion

### 3.1. Comparative Analysis of Turning Deformation with Typical Titanium Alloy Materials

**Figure 5:**
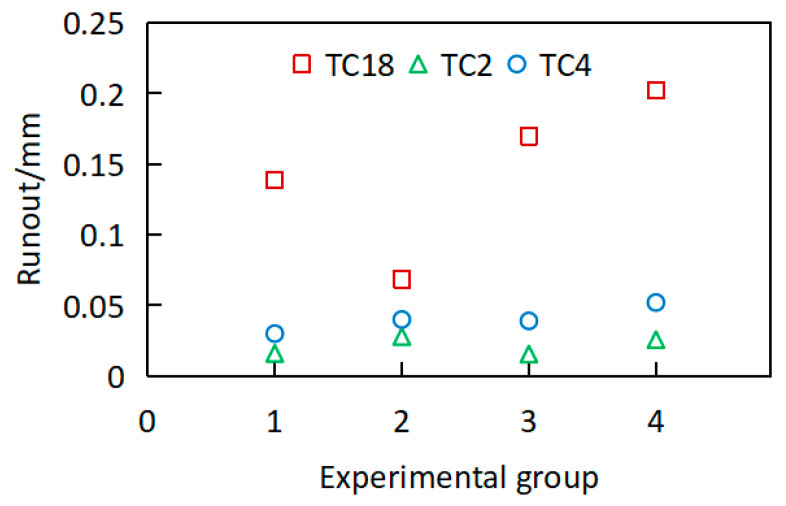
Comparison of deformation of three typical titanium alloys after turning.

To compare the turning characteristics of TC18 with those of other common titanium alloys, TC2 and TC4 alloy forgings were also included in this turning comparative study. The structural dimensions of the long-axis workpieces for all three materials were identical and met the acceptance criteria. The cutting scheme employed was scheme No. 1 in Table 1. For each material, four workpieces were tested to minimize measurement errors. After turning, the deformation was measured, and the results are shown in Figure 5.

As shown in Figure 5, it is evident that the runout of TC2 and TC4 titanium alloy slender shafts can be maintained within 0.05 mm after turning, whereas the runout of TC18 titanium alloy slender shaft ranges from 0.058 to 0.2 mm, which is significantly higher than that of TC2 and TC4. This result demonstrates that, in contrast to TC2 and TC4, TC18 exhibits substantial deformation after turning, leading to significantly higher difficulty in deformation control.

### 3.2. Analysis of the Variation Law of Residual Stress and Deformation

For precision machining, between-centers turning was employed to investigate the deformation of the workpiece following the removal of the top constraints at both ends after turning, as well as the deformation following stress relief annealing, through runout measurements. To minimize measurement errors, nine workpieces were selected for processing and subsequent testing.

**Figure 6:**
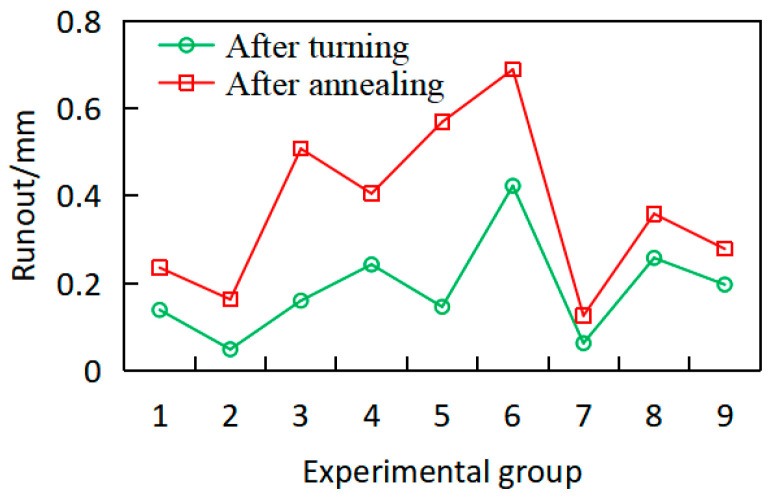
Comparison of runout of workpieces after precision machining and annealing.

The runout of the workpiece after precision machining and annealing is shown in Figure 6. After precision machining, the radial runouts of the workpiece are predominantly greater than 0.05 mm, with the maximum runout reaching 0.4045 mm. This phenomenon can be attributed to the reduced stiffness and the rebalancing of internal stresses of the workpiece after the turning process, which leads to increased deformation. After the annealing treatment, the workpiece deformation is further exacerbated, with the maximum radial runout reaching 0.6682 mm, resulting in the scrapping of some workpieces and the inability to complete subsequent processing. The reason is that, during turning, the radial allowance of the workpiece is removed unevenly, leading to significant and non-uniformly distributed internal stresses. However, during annealing, the residual stresses on the surface and within the workpiece are released under high temperature, causing a redistribution of internal stresses and further altering the deformation characteristics of the workpiece.

**Figure 7:**
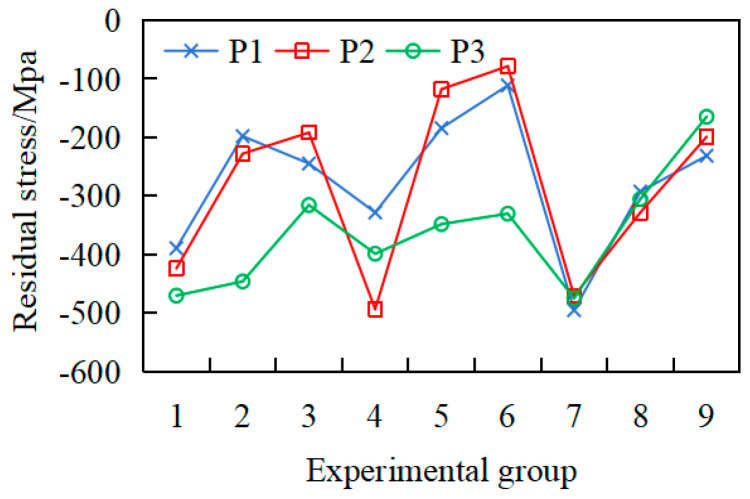
Average residual stress distribution of each section after precision machining.

To verify that the workpiece deformation originates from uneven stress distribution along the axial direction, residual stress measurements were conducted on the mid-sections of the first, second, and third axial segments, designated as P1, P2, and P3, respectively, as shown in Figure 1. Four equally spaced measurement points were selected along the circumferential direction of each cross-section for axial residual stress evaluation. The averaged values for each cross-section were calculated for comparative analysis, as shown in Figure 7.

From Figure 7, it can be seen that after precision machining, the surface residual stresses of the workpiece exhibit negative values, indicating compressive stresses. The compressive stress distribution ranges from −78.5 to −493.7 MPa, with considerable variability. Significant difference in residual-stress values are observed across different cross-sections of the same workpiece. For instance, in workpiece 2, P1 = −200 MPa, whereas P3 = −475 MPa, demonstrating non-uniform axial distribution of residual stresses on the workpiece surface, which may induce workpiece deformation. Although each workpiece is processed using identical machining parameters, significant differences in residual stresses are present. The observed phenomenon arises from significant axial heterogeneity in initial residual stress distribution during material fabrication, coupled with post-machining stress redistribution, ultimately leading to pronounced surface stress variations.

**Figure 8:**
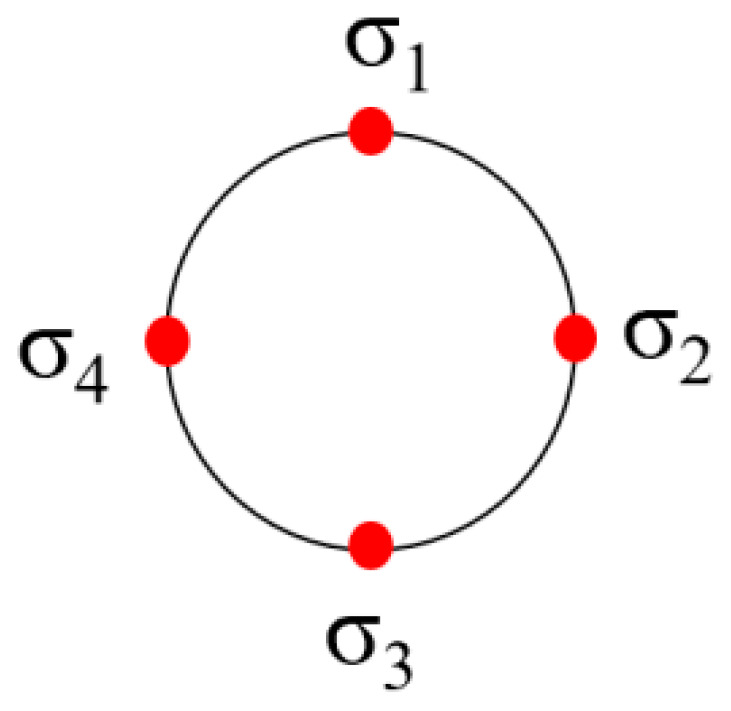
Selection of measurement points.

**Figure 9:**
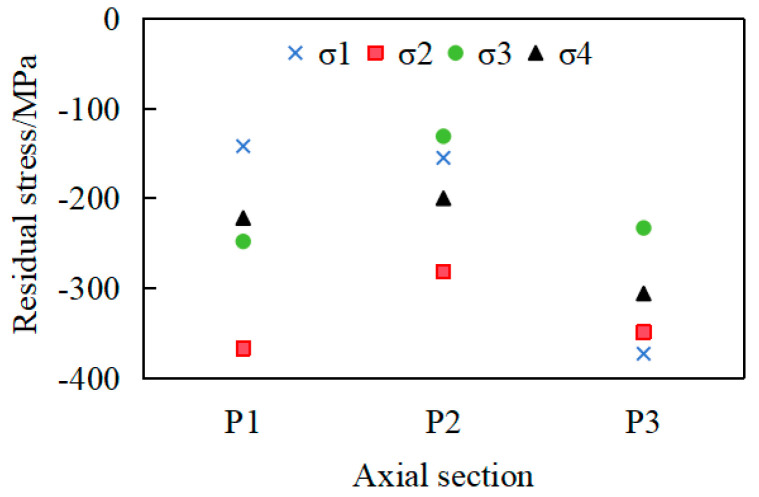
Distribution of residual stress along the circumferential direction in different cross-sections.

In order to compare the residual stress values on the same section, the third group of workpieces was randomly selected, with the section at the middle of each axial segment chosen for residual stress measurement. On each cross-section, four measurement points are uniformly distributed along the circumferential direction, designated as σ_1_, σ_2_, σ_3_, and σ_4_, as shown in Figure 8, and the measurement results are shown in Figure 9.

The results demonstrate that the residual-stress values exhibit significant variations across different measurement points within the same section. For instance, at section P1, the maximum residual stress reaches −367 MPa, while the minimum is −142 MPa. Such severe circumferential non-uniformity in residual-stress distribution leads to warping and substantial deformation in long-axis workpieces with low stiffness.

### 3.3. The Influence of Turning Scheme on Deformation and Optimization Improvement

Based on the above experimental findings, it was concluded that TC18 titanium alloy demonstrates high sensitivity to stress and strain during turning operations, particularly when machining slender forged shafts. After annealing treatment, the maximum runout of the precision-machined workpiece reached 0.6682 mm. To address this, a semi-precision machining step with a 0.8 mm allowance was introduced prior to the annealing and final precision machining processes to ensure dimensional accuracy.

In order to investigate the effects of different precision machining schemes on the deformation and surface quality of the workpieces, the between-centers turning scheme was adopted to conduct a single-factor cutting experiment, where the feed rates were 0.05 mm/r, 0.1 mm/r, 0.15 mm/r, and 0.3 mm/r; the cutting speed values were 640 r/min, 800 r/min, 1000 r/min, and 1500 r/min; and the cutting depth values were 0.05 mm, 0.1 mm, 0.2 mm, and 0.4 mm. Due to the high sensitivity of the deformation degree during between-centers turning of slender shaft workpieces with eccentric structures to the thrust force of the center, instability frequently occurs. Therefore, the thrust force was varied as a variable, with the percentage of the rated thrust force of the machine tool as the variable factor, 6%, 10%, 15%, and 40%.

Furthermore, in order to determine the optimal turning parameters, workpiece vibration during machining must be taken into account. Specifically when inappropriate parameters are selected, workpiece vibration intensifies due to the combined effects of the thrust force of the center and centrifugal force. Consequently, surface roughness Ra was incorporated as an additional evaluation criterion, with measurement results presented in Table 3.

The experimental results from groups 1, 2, 3, and 4 in Table 3 demonstrate that when the thrust force of the center exceeds 15% of rated thrust force, the radial runout of the workpiece exhibits a significant increase, resulting in dimensional deviations and elevated surface roughness. The reason is that the low stiffness of the slender shaft workpiece intensifies its vibration under excessive thrust force, leading to increased runout and roughness. However, when the thrust force is reduced to 6%, the workpiece roughness still increases due to inadequate clamping, which fails to sufficiently dampen the vibrations. Additionally, the data from groups 2, 8, 9, and 10 reveal that when the cutting speed is elevated to 1500 r/min, the peak workpiece runout reaches 0.345 mm. This phenomenon is attributed to an eccentric geometry in the mid-section of the workpiece. At higher rotational speeds, the augmented centrifugal force intensifies machining vibrations, consequently increasing both workpiece deformation and surface roughness.

The experimental results from groups 2, 5, 6, and 7 demonstrate that when the cutting depth increases to 0.4 mm, the runout of the workpiece exhibits an increasing trend. The reason is that as the cutting depth increases, the workpiece is subjected to increased radial cutting force, and the radial compression of the workpiece increases, resulting in increased deformation. Compared with the surface roughness in groups 2 and 5, when the cutting depth reaches 0.2 mm in group 6, the roughness value exhibits a decreasing trend. The reason is that when the titanium alloy is cut, the depth of the hardened layer is about 0.2 mm [17]. When the cutting depth exceeds the depth of the hardened layer of the workpiece, the vibration decreases. From the data of groups 2, 11, 12 and 13, it can be seen that when the feed rate increases to 0.3 mm/r, the runout of the workpiece increases and the roughness increases significantly, and the reason is that as the feed rate increases, the cutting force increases, causing an increase in workpiece vibration.

In summary, to minimize workpiece deformation and ensure surface quality during turning, between-centers turning should be employed, with strict control of the turning parameters. Optimal results for long-axis workpieces, characterized by minimal deformation and good surface quality, are achieved when the rotational speed is set to 640–800 rpm, the feed rate to 0.05–0.1 mm/r, the cutting depth to 0.1 mm, and the thrust force of the center to 10% of the rated value. Additionally, given the significant deformation that workpieces undergo after annealing, when utilizing the between-centers turning method, to ensure the positional and dimensional accuracy of the center holes, center holes should be refined or remachined if the billet length permits, guaranteeing concentricity between the center holes on both ends.

Based on the experimental results, exponential models for roughness *Ra* and runout Rt are independently established, with their expressions formulated as follows:

(1) $$Ra\;(Rt)=C\cdot \nu^{k}\cdot a_{p}^{m}\cdot f^{n}\cdot F^{q}$$

The correction coefficient of ***C*** depends on the processing material and cutting conditions; *ν*, *a_p_*, *f*, and *F* represent the rotational speed, cutting depth, feed rate, and the thrust force of the center, respectively. Items *k*, *m*, *n*, and *q* are constants associated with the cutting parameters. Equation (1) is subjected to linear transformation and linear regression analysis to calculate the linear regression correlation coefficient. The fitted parameter values are then substituted into the formula to obtain the exponential model (2) of surface roughness Ra, and the model is subjected to the F-test.

(2) $$Ra=0.0077\cdot \nu^{1.3142}\cdot a_{p}^{0.1684}\cdot f^{0.7370}\cdot F^{0.6752}$$

Based on the variance analysis of the empirical formula in Table 4, the calculated F-statistic is 12.30944 at a significance level of α = 0.05. With F (p, n − p – 1) = F_0.05_ (4, 8) = 3.84, with the model’s F-value exceeding this critical value, it is concluded that the model is statistically significant.

Furthermore, the exponential model for runout ***Rt*** is formulated as follows:

(3) $$Rt=2.7730\cdot \nu^{2.1997}\cdot a_{p}^{0.5222}\cdot f^{0.5347}\cdot F^{0.5956}$$

Based on the variance analysis of the empirical formula in Table 5, the calculated F-statistic is 4.488708 at a significance level of α = 0.05. With F (p, n − p – 1) = F_0.05_ (4, 8) = 3.84, and the model’s F-value exceeding this critical value, it is concluded that the model is statistically significant.

### 3.4. Influence of Heat Treatment Scheme on Deformation and Optimization and Improvement of the Scheme

#### 3.4.1. The Influence of Heat Treatment Scheme on Deformation and Residual Stress

As shown in Figure 6, the workpiece deformation exhibits a marked increase after annealing compared to the turning process, exceeding the machining allowance. Insufficient allowance may result in workpiece rejection. This deformation change is attributed to annealing conditions such as temperature and holding time. If residual stresses persist within or on the workpiece surface during annealing, they may adversely affect subsequent chromium plating and service performance, potentially inducing defects such as cracking.

In order to further verify whether the annealing conditions can effectively remove residual stress, different heat treatment schemes were designed, namely changing the annealing temperature and holding time, changing the heat treatment temperature from 600 to 680 °C, and the holding time from 1 to 3 h. The annealing conditions are established as shown in Table 6. Nine sets of workpieces were selected for annealing tests to be conducted at different temperatures and holding times. In order to ensure the accuracy of the test results, three workpieces were selected for each set and tested using a single-factor experimental method.

**Figure 10:**
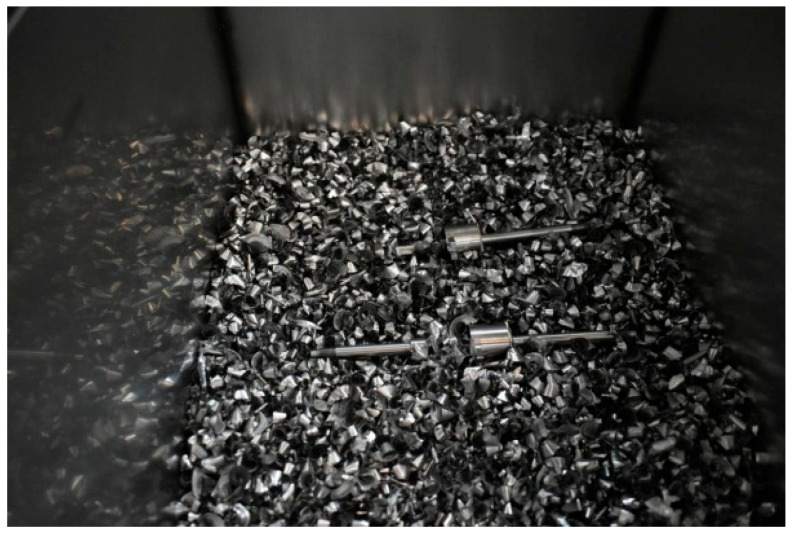
Using titanium chips to fully cover the workpiece in a box.

Prior to annealing, the workpieces were machined using the optimized turning scheme, achieving a runout < 0.05 mm. To prevent oxidation of precision surfaces during annealing, all workpieces were processed in a vacuum furnace with complete covering in titanium chips within dedicated containers, as shown in Figure 10. Post-treatment cooling was conducted under argon atmosphere, with the titanium chip containers undergoing controlled air cooling to room temperature prior to workpiece removal.

**Figure 11:**
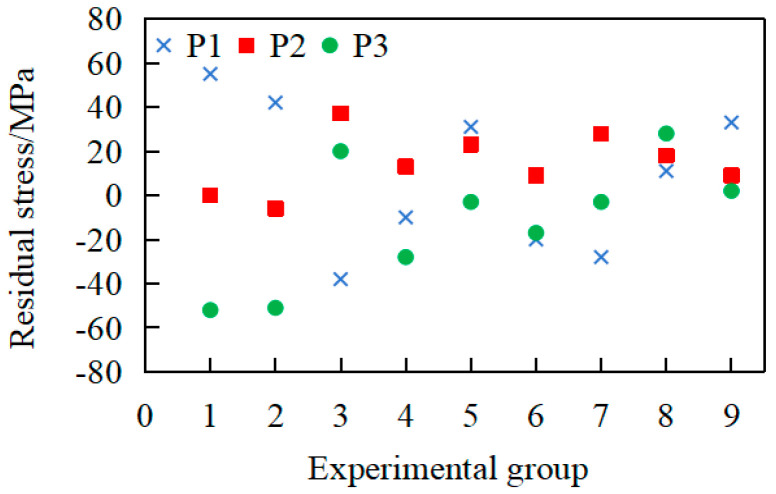
Residual-stress distribution under different heat treatment conditions.

The residual stress of the workpiece under the aforementioned working conditions was measured employing the identical detection scheme illustrated in Figure 4c. The average residual-stress values for each shaft section (P1, P2, and P3) were obtained, and the statistical results are shown in Figure 11.

As shown in Figure 11, under the processing conditions for groups 1–3, the post-annealing surface residual stresses exhibited broad distributions exceeding ±50 MPa. From group 4 onward, the residual stresses stabilized at approximately ±30 MPa, indicating enhanced stress-removal efficacy. Residual stresses are considered effectively eliminated when their post-annealing distribution falls within ±50 MPa. It can be considered that the residual stress was effectively removed. These experimental results show that the selected annealing protocol (640 °C for 1.5 h) successfully eliminated both machining-induced surface residual stress and forging-inherited internal stress. Therefore, in order to ensure the removal effect of residual stresses and improve the heat treatment efficiency, an annealing temperature of 640 °C with a 1.5 h holding time was applied in subsequent experimental processes.

**Figure 12:**
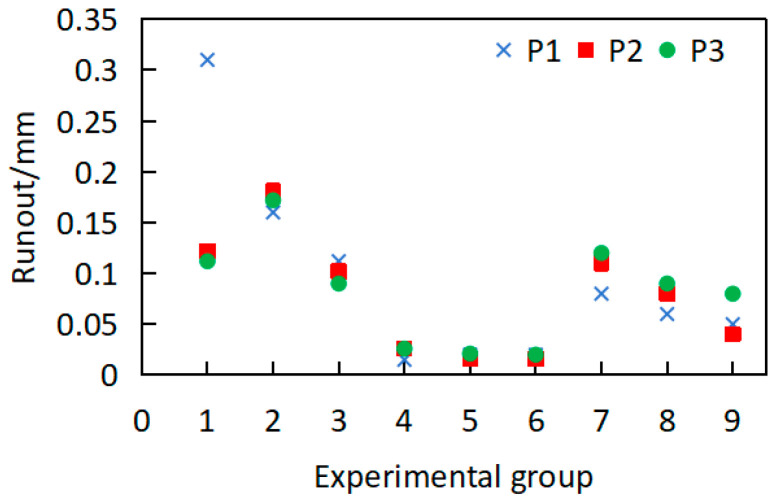
Runout distribution under different heat treatment conditions.

Concurrently, workpiece deformation under the above working conditions was measured, as shown in Figure 12. The runout of workpieces following precision machining and re-annealing treatment is shown in Figure 12. Owing to the optimized turning parameters, the post-annealing runout is predominantly confined to 0.02–0.1 mm, representing a marked reduction compared to the first annealing. The workpiece warpage deformation exhibits substantial improvement. The reason is that residual stress was fully released after annealing following semi-precision machining; then, during precision machining, the reduced and more uniform radial material removal leads to a more homogeneous residual-stress distribution, thereby resulting in significantly diminished workpiece deformation after the secondary annealing process. However, it can be seen that the runout of individual workpieces still increases significantly compared to the workpiece after turning, with a maximum runout of 0.31 mm. The reason for this is that the residual-stress distribution of individual workpieces still exists after cutting, and the uneven stress release after annealing leads to increased deformation.

However, the runout distribution still demonstrates excessive dispersion, as shown in Figure 12. which is not conducive to final size control. In the above deformation situation, subsequent processes such as sandblasting, diffusion annealing, milky white chromium plating, chrome plating, and grinding will be carried out, and the risk of increased runout of the workpiece is still high. Therefore, further optimization of the heat treatment scheme is needed.

#### 3.4.2. Optimization and Improvement of Heat Treatment Scheme

**Figure 13:**
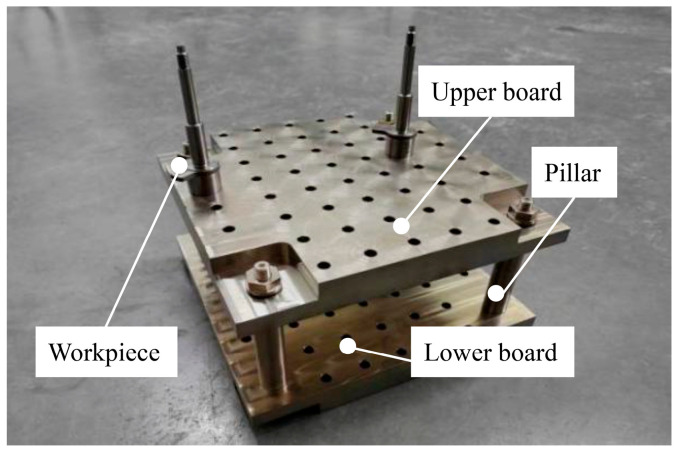
Vertical placement of workpieces.

During the heat treatment process, titanium chips are utilized to cover the workpieces for high-temperature oxidation prevention; the placement method adopted is an unconstrained placement methodology (i.e., arbitrary orientation coverage with titanium chips). Theoretically, the minimal mass of titanium chips suggests negligible influence on workpiece deformation. However, for precision-sized slender shaft workpieces characterized by limited structural rigidity and compromised deformation resistance, the combined effects of titanium chip distribution and gravitational forces become non-negligible in elevated temperature environments. To verify the influence of the placement orientation of the workpiece on deformation, a dedicated fixture was engineered to maintain strict vertical alignment during processing, as shown in Figure 13. This fixture–workpiece assembly was integrally embedded within titanium chips inside the containment box. Based on the conclusion of Figure 10, the workpiece was annealed at 640 °C for 1.5 h, and the runout was precisely measured to study the effect of workpiece placement methods on the runout after heat treatment

**Figure 14:**
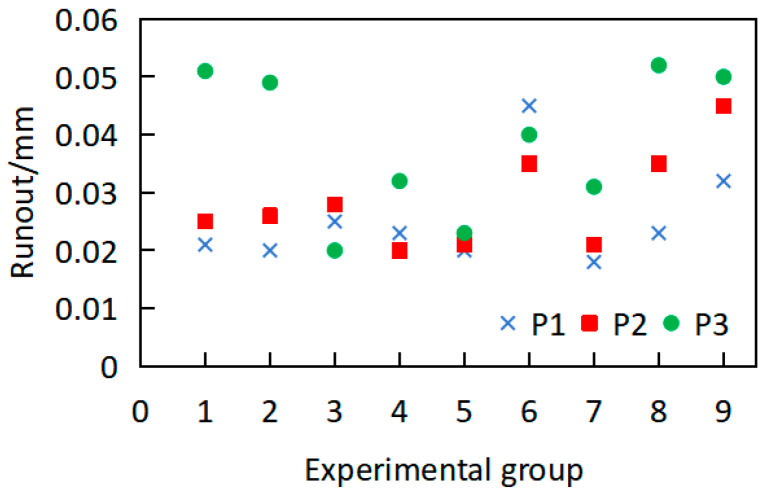
Runout when the workpiece is placed vertically.

The runout distribution following adjustment of the workpiece annealing placement is shown in Figure 14. Comparative analysis with the horizontal free placement in Figure 12 indicates that transitioning the workpiece from arbitrary horizontal orientation to vertically constrained placement using a dedicated fixture achieves the following outcomes: runout values are distributed within 0.018–0.051 mm, the distribution range is significantly narrowed, and runout magnitude decreases by approximately 50%, basically satisfying the technical requirement of ≤0.05 mm. This demonstrates that modifying the heat treatment methodology from horizontal free placement to vertically constrained placement effectively reduces thermal deformation and mitigates gravitational influences. Consequently, for slender shaft workpieces characterized by low stiffness and high-precision requirements, heat treatment placement orientation constitutes a critical factor affecting deformation, necessitating specialized vertical fixturing to minimize post-annealing distortion.

### 3.5. Analysis of Grinding Effect Verification

**Figure 15:**
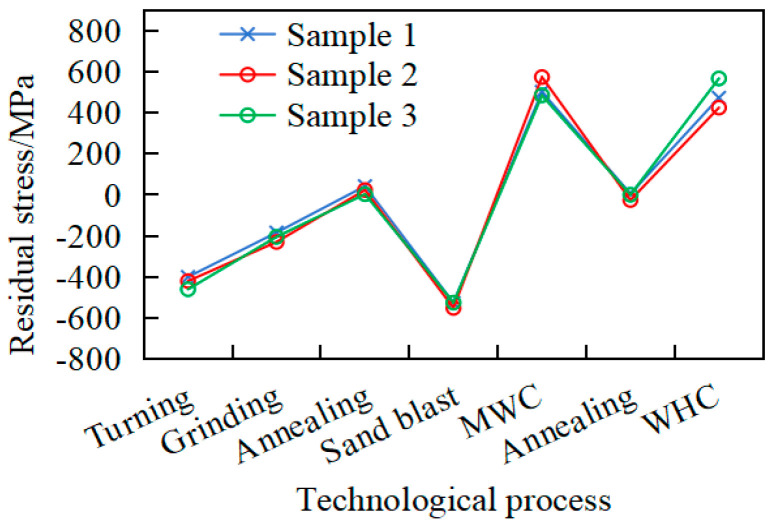
Changes in residual stress under different processes.

**Figure 16:**
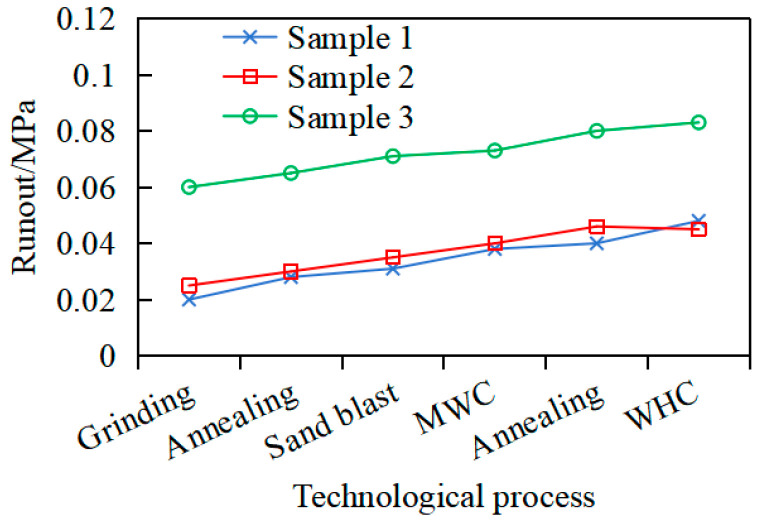
Changes in runout under different processes.

After processing, the workpiece undergoes a sequence of processes comprising grinding, annealing, sandblasting, milky white chromium (MWC) coating, vacuum diffusion annealing, hard chromium (WHC) coating, and final precision grinding. To validate the efficacy of the optimized processing and heat treatment scheme in achieving final grinding compatibility and surface integrity, three workpieces with initial maximum runouts of 0.02 mm, 0.02 mm, and 0.06 mm were strategically selected for full-scale process validation. And the residual stress and deformation were measured, as shown in Figure 15 and Figure 16.

As shown in Figure 15, the surface residual stress exhibits substantial fluctuations during the manufacturing process, with repeated alternations between compressive and tensile states. After turning, the surface residual compressive stress reaches −400 MPa, which is subsequently reduced to −200 MPa following the initial grinding stage. This stress evolution arises primarily from the higher material removal rate from turning compared to that from grinding, where the more severe plastic deformation induces greater compressive residual stresses.

Sandblasting can significantly increase the value of surface roughness and improve the adhesion of coatings, but also increase the residual compressive stress on the surface to approximately −600 MPa. Furthermore, as shown in Figure 15, after grinding, the workpiece still needs to undergo two annealing processes, which effectively adjust its residual stress value to near 0 MPa, thereby eliminating the stress. During manufacturing processes, residual stress on the workpiece surface experiences dynamic transitions between compressive and tensile states, accompanied by substantial variations in stress magnitudes. Taking sample 3 as an example, during sandblasting and chrome plating, the residual stress value increases from −525 MPa to +566 MPa, which is not conducive to deformation control. Therefore, to mitigate workpiece deformation, beyond controlling the turning and heat treatment parameters, each process step requires stringent control. For instance, automated sandblasting equipment is recommended to ensure uniform stress distribution on the workpiece surface.

As shown in Figure 16, multiple annealing and electroplating processes will progressively exacerbate workpiece deformation. Specifically, the runout of sample 1 and sample 2 measures 0.045 mm and 0.048 mm, respectively, whereas sample 3 exhibits a significantly higher runout of 0.083 mm. This trend arises from cyclic residual stress generation and redistribution during manufacturing, with localized stress inhomogeneity inducing radial runout accumulation. Notably, the initial runout of workpiece 1 and workpiece 2 is relatively small, so they can ultimately meet the requirement of 0.05 mm runout. These findings suggest that strict control of machining-induced runout is critical to reserve adequate dimensional tolerance for subsequent processes, such as sandblasting.

**Figure 17:**
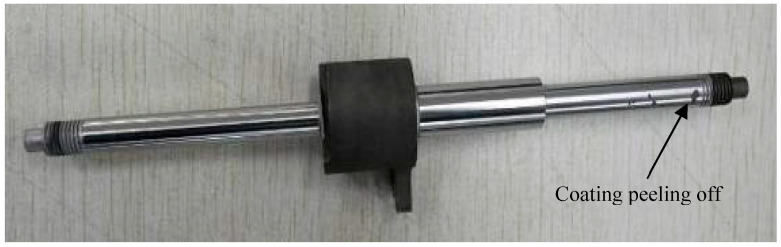
Localized detachment of chromium layer on workpiece after grinding.

Following chromium electroplating and subsequent grinding processes, samples 1 and 2 met specifications, whereas sample 3 failed due to localized coating detachment at its point of maximum radial runout. As shown in Figure 17, localized coating detachment occurred exclusively at the workpiece end. This phenomenon was attributed to warping deformation at this location, resulting in reduced coating thickness and inadequate interfacial bonding strength after grinding. Furthermore, as shown in Figure 15, the residual tensile stress on the surface of the workpiece after chrome plating was relatively high, which can promote microcrack initiation and propagation toward the coating–substrate interface, degrading interfacial adhesion, then coating detachment may occur when interfacial adhesion strength falls below the applied grinding forces.

**Figure 18:**
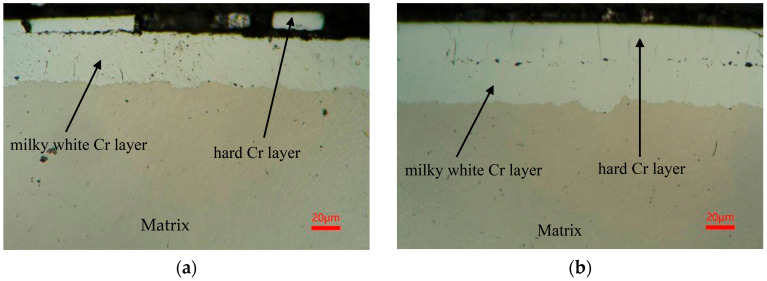
Metallographic structure after chrome plating and grinding. (**a**) Chromium layer peeling off; (**b**) chromium layer has not peeled off.

To further investigate the chromium layer microstructure after grinding and analyze the reasons for localized detachment of the chromium layer, samples 3 and 2 underwent wire electrical discharge machining, followed by mechanical polishing, electrolytic etching, and then were subjected to metallographic structure comparison observation, as shown in Figure 18.

From Figure 18, it is evident that the chromium layer surface is flat and smooth after grinding. In contrast, sample 3 exhibits localized detachment of the chromium layer, specifically at the interface between the hard chromium and milky chromium layers. The residual thickness of the hard chromium layer after grinding is approximately 10 μm, with localized detachment observed. Conversely, sample 2, featuring an initial radial runout of 0.02 mm, retained a coating thickness of about 30 μm after grinding, exhibiting uniform thickness distribution. When there was no detachment of the coating, and the appearance inspection showed no cracks, the product was qualified. Localized thinning of the coating or detachment occurs because the initial electroplating thickness is comparable, but significant workpiece runout leads to localized runout variations, causing thinning of the coating after grinding. When the thickness is less than 10 μm, the interfacial bonding strength between the hard chromium and milky chromium layers is insufficient to withstand the grinding shear forces, resulting in the detachment of the hard chromium layer. For workpieces with minimal radial runout, the grinding removal rate is uniform, ensuring uniform residual coating thickness. Therefore, it is essential to rigorously control workpiece deformation during processing to prevent the coating from becoming excessively thin or detaching.

**Figure 19:**
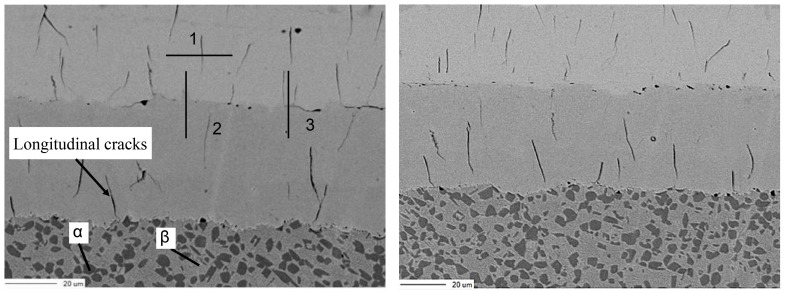
Microstructure and morphology.

To further analyze the microstructure and morphology of the substrate and coating and investigate the mechanisms underlying localized coating detachment, backscattered electron imaging and electron probe microanalyzer elemental mapping were conducted on the sample, with the corresponding results presented in Figure 19.

**Figure 20:**
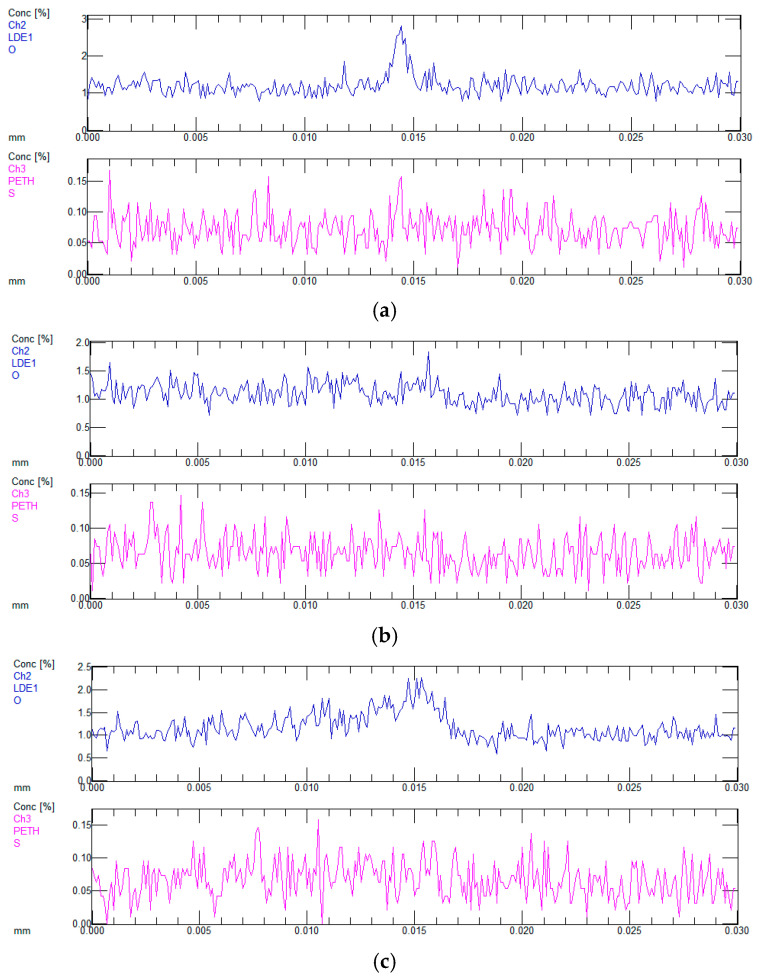
Distribution of O and S elements at different positions. (**a**) Component analysis of path 1; (**b**) component analysis of path 2; (**c**) component analysis of path 3.

As shown in Figure 19, longitudinal microcracks are distributed within both the milky white chromium layer and the hard chromium layer, with transverse microcracks predominantly observed at their interface. Per the acceptance criteria, when the bonding force is guaranteed, the presence of non-penetrating microcracks in the coating is permissible. The formation of these microcracks may be attributed to substantial residual tensile stresses developed on the surface during electroplating. Surface microdefects in the coating, particularly pores, may serve as stress concentration sites, initiating cracking under tensile stress. During vacuum diffusion annealing, hydrogen desorption from the chromium layer induces localized volumetric contraction, which amplifies stress concentration and consequently promotes crack propagation. To characterize the compositional variations adjacent to microcracks, elemental composition analysis was conducted using an electron probe at selected locations and along defined trajectories, as shown in Figure 20.

Scanning path 1 in Figure 20a traverses the longitudinal crack within the coating, while scanning path 2 spans the continuous interface between the milky white chromium layer and the hard chromium layer. Scanning path 3 crosses the transverse crack located at the coating connection. As shown in Figure 19 and Figure 20, paths 1 and 3 intersect longitudinal and transverse cracks, respectively, where significant enrichment of O and S elements is observed. In contrast, path 2 passes through a crack-free interface without detectable enrichment of O and S elements. Given the elevated concentrations of O and S in the electrolyte, it can be inferred that microcracks develop during the electroplating process.

In summary, improper control of electroplating parameters, such as current density and solution ratio, can result in the formation of longitudinal cracks and transverse detachment within the coating. Additionally, during workpiece manufacturing, excessive runout leads to thinning of the coating after grinding, reducing the interfacial bonding strength, and directly causing localized coating detachment. Therefore, in the manufacturing process, the runout of the workpiece must be strictly controlled to <0.05 mm to ensure coating integrity.

## 4. Conclusions

In this paper, the residual stress evolution and deformation behavior of TC18 titanium alloy slender shafts during precision machining and heat treatment processes were investigated. Furthermore, based on the deformation patterns and stress distribution laws, the turning and heat treatment strategies were optimized and improved. The main conclusions are as follows:

(1) Through the preparation of qualified forging blanks followed by annealing treatment, TC18 titanium alloy slender shafts were successfully precision manufactured. The deformation analysis revealed significant warpage after turning, with a pronounced increase in deformation following subsequent annealing. Surface residual-stress measurements indicate compressive stresses ranging from −78.5 to 493.7 MPa after turning, exhibiting substantial axial fluctuations.
(2) Turning experiments on slender shafts were conducted to investigate the effects of feed rate, cutting speed, depth of cut, and axial thrust force of machine tool center on machining deformation and surface roughness. Considering the workpiece’s eccentric structure and low overall stiffness, optimal machining parameters are determined as follows: cutting speed of 640–800 r/min, feed rate of 0.05–0.1 mm/rev, depth of cut of 0.1 mm, and clamping force at 10% of the rated value. These parameter combinations resulted in minimal workpiece deformation and optimal surface quality.
(3) Heat treatment experiments were performed on precision-turned TC18 slender shafts at varying temperatures and holding durations, with subsequent residual-stress measurements. The results demonstrate that residual stresses could be effectively eliminated within the temperature range of 640–680 °C and a holding time of 1–3 h. For low-stiffness slender shaft workpieces, the orientation during heat treatment significantly influences workpiece deformation, and the vertical fixturing method is found to reduce deformation by approximately 50% compared to conventional approaches.
(4) The manufacturing of TC18 titanium alloy long shafts involves a series of processes including machining, heat treatment, and electroplating. A minimal initial runout is essential to guarantee coating uniformity after grinding. Process controls must ensure stress uniformity during machining and sandblasting operations. Furthermore, electroplating parameters require strict regulation to reduce microcrack formation and prevent chromium layer detachment during grinding.

## Figures and Tables

**Table 1:** Chemical composition of TC18 titanium alloy.

Element	Mo	Cr	Si	N	C	Fe	V	Al
Content (Wt%)	4.0~5.5	0.5~1.5	<0.15	<0.05	<0.1	0.5~1.5	4.0~5.5	4.4~5.7

**Table 2:** Turning processing scheme and parameters.

Serial Number	Processing Method	Speed r/min	Feedrate mm/r	Cutting Depth/mm	Clamping Method	Diameter Allowance/mm
1	Rough machining	350	0.25	1.2	One end clamped, one end pressed tightly	0.4
2	Precision machining	640	0.15	0.2	Top to bottom at both ends	0.4

**Table 3:** Comparison of the influence of different turning schemes on deformation and surface quality.

Number	Feed/(mm/r)	Cutting Speed/(r/min)	Cutting Depth/mm	Thrust Force/%	Runout /mm	Roughness /μm
1	0.05	640	0.05	6%	0.068	0.512
2	0.05	640	0.05	10%	0.038	0.503
3	0.05	640	0.05	15%	0.072	0.614
4	0.05	640	0.05	40%	0.123	1.56
5	0.05	640	0.1	10%	0.05	0.521
6	0.05	640	0.2	10%	0.068	0.481
7	0.05	640	0.4	10%	0.172	0.964
8	0.05	800	0.05	10%	0.048	0.611
9	0.05	1000	0.05	10%	0.102	0.935
10	0.05	1500	0.05	10%	0.345	1.68
11	0.1	640	0.05	10%	0.043	0.75
12	0.15	640	0.05	10%	0.052	1.48
13	0.3	640	0.05	10%	0.174	1.82

**Table 4:** Variance analysis of surface roughness prediction model.

Source of Variance	df	SS	MS	F	Significance F
Regression analysis	4	2.642014	0.660504	12.30944	0.001695
Residual	8	0.429266	0.053658		
Total	12	3.071281			

**Table 5:** Variance analysis of runout prediction model.

Source of Variance	df	SS	MS	F	Significance F
Regression analysis	4	3.613672433	0.903418	4.488708	0.034001
Residual	8	1.610117061	0.201265		
Total	12	5.223789495			

**Table 6:** Single-factor experimental design for heat treatment.

Experimental Group	Temperature/°C	Holding Time/h
1	600	1
2	600	1.5
3	600	3
4	640	1
5	640	1.5
6	640	3
7	680	1
8	680	1.5
9	680	3

## Data Availability

The original contributions presented in this study are included in the article. Further inquiries can be directed to the corresponding author.
